# Survival of an 80-Year-Old Male With a Successful Split-Thickness Skin Graft for End-Stage Necrotizing Fasciitis: A Case Report

**DOI:** 10.7759/cureus.25829

**Published:** 2022-06-10

**Authors:** Mashood Iqbal, Ahsan Arshad, Jahanghir Syed, Amna Saleem, Abdus Salam Khan, Shayan Iqbal Khan, Uzzam Ahmed Khawaja

**Affiliations:** 1 Internal Medicine, Jinnah Medical College Hospital, Karachi, PAK; 2 Internal Medicine, Shaikh Khalifa Bin Zayed Al-Nahyan Medical and Dental College, Lahore, PAK; 3 Internal Medicine, Liaquat University of Medical and Health Sciences, Hyderabad, PAK; 4 Neurological Surgery/General Surgery, Jinnah Medical and Dental College, Karachi, PAK; 5 Orthopedic Surgery, Jinnah Medical and Dental College, Karachi, PAK; 6 Internal Medicine, Jefferson Health, Abington, USA; 7 Internal Medicine, Jinnah Medical and Dental College, Karachi, PAK; 8 Clinical and Translational Research, Dr. Ferrer BioPharma, Aventura, USA

**Keywords:** necrotizing fasciitis, wound debridement, skin grafting, poor prognosis, limb amputation

## Abstract

Herein, we report a case of an 80-year-old male who was diagnosed with a fatal condition known as necrotizing fasciitis. This devastating soft tissue infection can cause profound damage to multiple tissue planes. Despite its etiology being multifactorial, impaired immunity with increasing age weighs in as the most significant. We intend to shed light on its detrimental clinical features and how we managed to treat the patient both conservatively and surgically. Through our case findings and management plan, we hope this case to be of clinical value and knowledge to clinicians to better diagnose and treat the deleterious condition.

## Introduction

Necrotizing fasciitis has been regarded as a soft tissue infection in an area of the body leading to rapid progressive destruction of the underlying tissues and fascia involving the lower limbs and the trunk [1,2]. Its occurrence requires prompt medical and surgical intervention to prevent fatal outcomes or amputation [3]. Interestingly, many cases have been reported regarding its dreadful course and a grim overall survival rate; however, the reconstruction of the affected tissue via a successful skin grafting modality has been delineated rarely in the elderly subset.

Various poor prognostic risk factors have been highlighted, including immune-compromised age group, alcoholics, and comorbidities including diabetes [4]. The latter has also been clearly labeled as a clinical predictor of limb amputation in a cohort study, thereby emphasizing its huge impact on disease progression [5]. Extremes of age have been proven to hinder the immune capacity of an individual to fight infections; hence, a poor prognosis is noted [6]. The mortality and morbidity ratio of the condition has been explicably high where the disease has been entailed to be diagnosed at a later stage, where in most cases, the effective treatment window passes away, rendering a futile overall treatment outcome [3].

Management of the morbidity appears to involve the surgical debridement of the underlying necrotic tissues and fascia [2]. Wound debridement may occur in a series of episodes with the patient being managed under close observation with adequate IV broad-spectrum antibiotic coverage [2,7].

We report a case of an elderly 80-year-old male patient with end-stage necrotizing fasciitis of the forearm and its successful treatment and anatomical restoration.

## Case presentation

**Figure 1:**
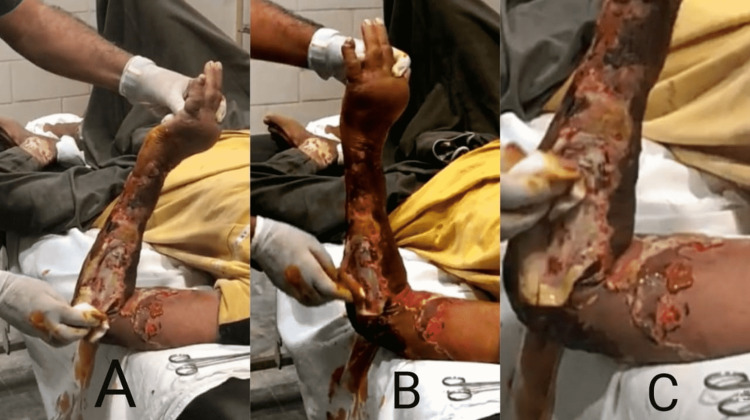
Forearm fasciitis with the destruction of the skin, underlying fascia, and multiple soft tissue defects with necrotic purulent discharge.

An 80-year-old male patient, professionally a retired goldsmith by occupation, with no known comorbidities presented to our emergency department with excruciating painful left forearm sloughing and erosion of superficial skin and underlying fascia, necrotic foul-smelling wound discharge, and prominent overall destruction of the forearm anatomy extending up to the cubital fossa (Figure 1).

The wound size was approximated to 30 x 15 cm with the patient displaying a septic look, lethargy with fever, and poor hydration status. According to the patient’s history of presenting illness, he was in a usual state of health two weeks ago when he injured his left forearm leading to an initial 2 x 2 cm open wound. The patient self-medicated himself at home with topical antibiotic application with the eventual poor progression of wound healing ultimately engulfing the entire forearm.

Acute kidney injury (AKI) was also identified due to an overall dehydrated status that was also witnessed in his laboratory parameters, as illustrated in Table 1 alongside other inflammatory markers indicative of chronic infection. A tissue biopsy could not be performed due to the lack of funds. Cultures from the wound revealed tazobactam-sensitive *Escherichia coli* and *Proteus mirabilis*.

**Table 1:** Remarkable laboratory parameters with reference ranges.

Laboratory parameters	Actual value	Reference range
Creatinine	1.6 mg/dL	0.7-1.3 mg/dL
White blood cell count	18,000 cells/mm^3^	4,000-11,000 cells/mm^3^
Erythrocyte sedimentation rate	175 mm/hour	<20 mm/hour
C-reactive protein	350 mg/L	<10 mg/L

The patient was managed accordingly with culture-sensitive intravenous antibiotics alongside three sessions of aggressive surgical debridement of circumferential skin tissue necrosis with infected superficial fascia for a total period of three weeks. The patient was initially given injectable Tanzo 4.5 g IV three times a day with further dose adjustment as per creatinine clearance function. Later, leflox injection of 750 mg IV once a day was added.

Management of AKI secondary to dehydration was as follows: (a) stringent 24 hours intake and output (I/O) charting with Foley output monitoring; (b) continuous hydration with 0.9% NaCl at 50 ml/hour IV on-flow with 250-300 ml 0.9% NaCl boluses as per need; (c) due to aggressive debridement sessions, packed cell volume (PCV) blood transfusions were done nearly five to six times to maintain hemoglobin at 10 g/dl for optimum wound healing; (d) oral encouragement of fluid intake per shift with daily urea, creatinine, and electrolytes (UCE) repeated; (e) daily UCE monitoring was done with gradual resolution of AKI accordingly; and (f) Ensure supplement was also started with two scoops two to three times per day.

**Figure 2:**
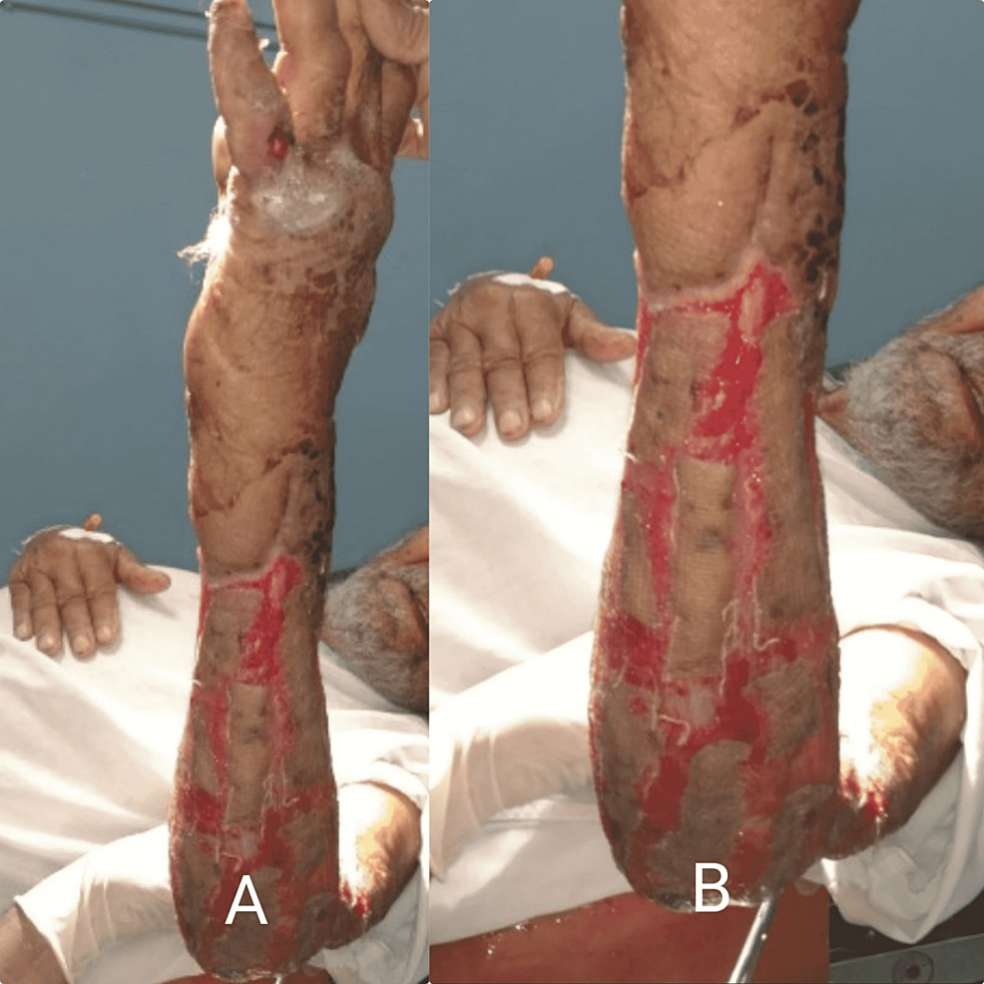
Three weeks post-split-thickness skin graft application showing healing with surrounding granulation tissue visualized.

From the fourth week, a split-thickness skin graft was taken bilaterally from the anterior thighs and applied evenly on the forearm with Sofra-Tulle dressings. Three weeks post-split-thickness skin graft application, the patient was followed for multiple dressing sessions with remarkable improvement in skin restoration (Figure 2).

**Figure 3:**
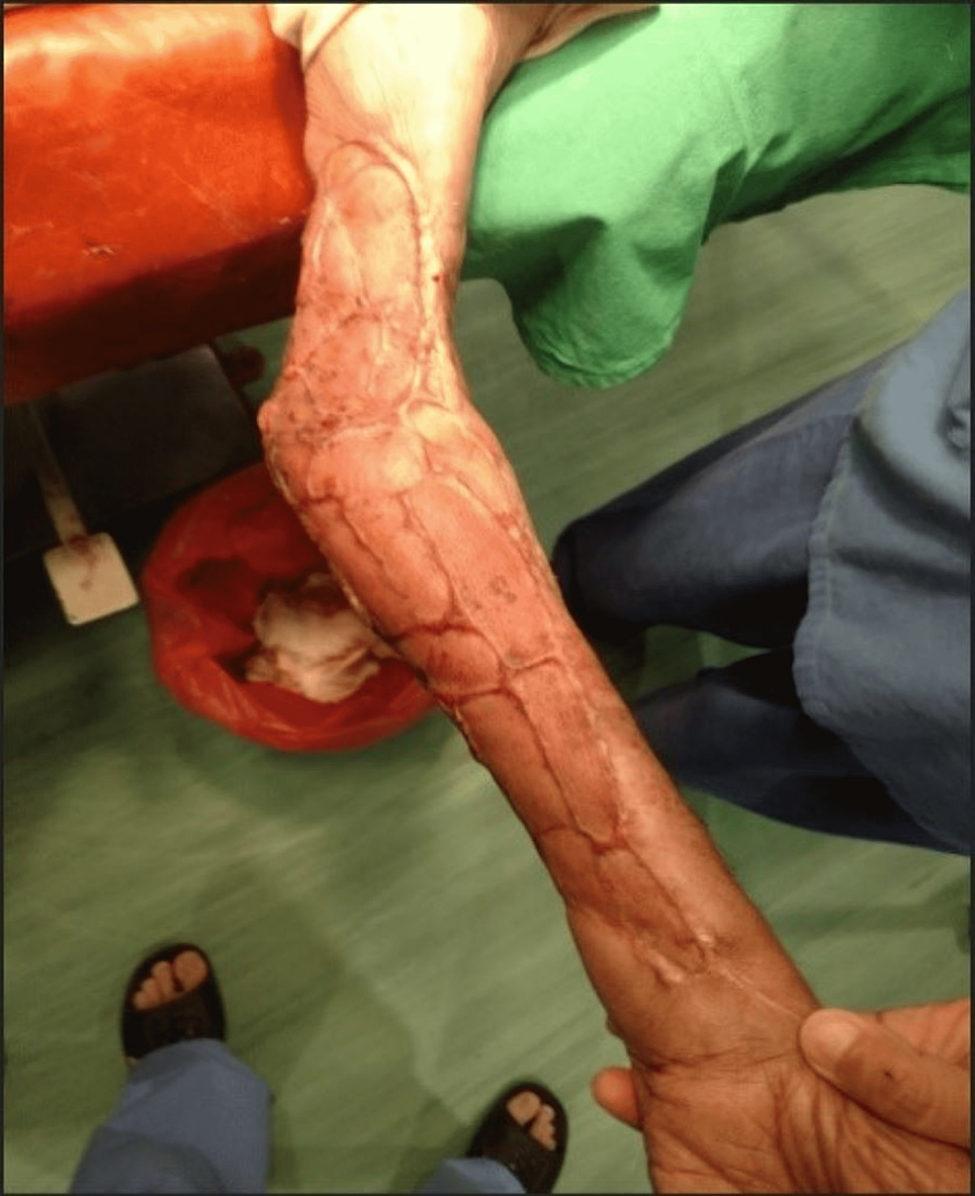
Six to eight weeks post-split-thickness skin application results in full healing with minimal granulation tissue visualized. The majority of the anatomical structure is restored.

The patient was followed further till six to eight weeks post grafting procedure and was found to have the majority of the anatomical restoration of the left forearm with no major complications encountered (Figure 3).

## Discussion

Necrotizing infections have been clearly labeled as life-threatening morbidity with a mortality rate as high as 76% described in a study, preferably involving the superficial fascia [4]. Nevertheless, having a grave outcome, it can be managed well if diagnosed at an earlier stage with an effective treatment strategy [8]. The concept of end-stage necrotizing fasciitis emphasizes the need for early intervention through surgical and medical modalities as the morbidity claiming itself to be lethal may hamper the chances of overall survival and ultimate loss of limb function and amputation when involved accordingly [5].

Manifestations of the condition majorly involve the extremities and evolve around an initial trauma in a healthy individual [4,9]. A retrospective study conducted from 1997 to 2002 concluded the scarcity of the symptoms to attain a confirmative diagnosis of the ailment, thereby delaying the necessary medical intervention. Prior injudicious antibiotic treatment without the recognition of the underlying disease spectrum has also been labeled as having a masking effect on the severity of the morbidity involved [8]. The literature review barely presents any specific symptomatology to conclude the presence of necrotizing fasciitis. Excruciating pain with erythematous blistering is predominantly the primary key characteristic emphasized in disease manifestation [10].

Diagnosing necrotizing fasciitis has been a challenge involving multiple preliminary investigations including the culture of the microorganisms involved, clinical evaluation, and imaging modalities such as the use of plain radiography to determine the presence of gas in soft tissue [10,11]. However, the role of culture and sensitivity of the microbes in aiding the ultimate diagnosis presumes to predominate in the majority of cases encountered [2,9]. A mixture of microbes, polymicrobial infiltration of the tissue by *Streptococcus pyogenes*, and coagulase-positive staphylococci have been reported thoroughly in the majority of the cases encountered [2,12]. However, *E. coli *as a perineal fasciitis-causing microbe has also been encountered in a 60-year-old female. This depicts a multitude of organisms that have been positively correlated with necrotizing fasciitis infections highlighting the variety of microbes involved in the ailment [13].

Intriguingly, a concept of the Laboratory Risk Indicator for Necrotizing Fasciitis (LRINF) scoring system has been introduced by Narasimhan et al. and Wong et al., where it has also been regarded as a breakthrough adjunctive for early diagnosis of necrotizing fasciitis. Despite its inclusion as a preventive adjunct measure, its application remains unused in a majority of cases [14,15].

The prime objective to effectively manage the morbidity widely entails the idea of a truculent surgical debridement for an ultimate favorable prognosis [4,16]. Multiple sessions of extensive radical surgical intervention are obligatory to efficiently eradicate the infectious agent successfully. Since surgical debridement of soft tissues can cause an ultimate loss of the anatomical structure involved, a surgical reconstruction for defect coverage may eventually become mandatory leading to a much more complex physician task objective [10,17]. The use of simultaneous wide-spectrum antibiotic coverage and resuscitation efforts acquire the need for a tertiary care health center burdening the financial crisis subset; hence, it is important to consider the disease as a high-level suspicion when considering cellulitis and abscess as a differential diagnosis [3].

Despite an established surgical treatment method, no efficacious therapeutic strategy with a protocol for early diagnosis has been established. This leads to a disastrous outcome when the morbidity is diagnosed at a stage where death by multiple organ failure and septicemia is imminent [3,4].

## Conclusions

Diagnosing necrotizing fasciitis presents an exigent task on account of its reduced occurrence, hasty growth, and high mortality. It should always be surmised in immunocompromised patients and in those whose symptoms are inordinate to its clinical presentation. Prompt diagnosis and expeditious surgical intervention along with multidisciplinary care can significantly reduce the chance of devastating outcomes like loss of physical function and limb amputation.
